# Assessment of Body Morphometry to Classify Two Colombian Creole Pigs Using Statistical and Machine Learning Methods

**DOI:** 10.3390/life15050693

**Published:** 2025-04-24

**Authors:** Arcesio Salamanca-Carreño, Mauricio Vélez-Terranova, Pere M. Parés-Casanova, Paula A. Toalombo-Vargas, David E. Rangel-Pachón, Andrés F. Castillo-Pérez

**Affiliations:** 1 Facultad de Medicina Veterinaria y Zootecnia, Universidad Cooperativa de Colombia, Villavicencio 50001, Colombia; david.rangelp@campusucc.edu.co (D.E.R.-P.);; 2 Facultad de Ciencias Agropecuarias, Universidad Nacional de Colombia, Palmira 763531, Colombia; 3 Departamento de Bromatología, Universitat Oberta de Catalunya, 08018 Barcelona, Spain; ppares@uoc.edu; 4 Programa de Zootecnia, Escuela Superior Politécnica de Chimborazo, Riobamba 060150, Ecuador

**Keywords:** biodiversity, body measurements, classification, ethnological characteristics, genetic resources, native breed

## Abstract

Creole pigs (*Sus scrofa domestica*), descendants of Iberian breeds, possess significant genetic and cultural importance but are under-researched and at risk due to the dominance of improved breeds for commercial production. The aim of this study was to identify the most representative body morphometric measurements for the differentiation of two Creole pig breeds, using statistical and machine learning methods. A sample of “Casco de Mula” (*n* = 54) and San Pedreño (*n* = 30) Creole pigs, aged between 2 and 6 months, belonging to seven traditional farms located in the department of Meta (Colombia), was studied. A total of 14 morphometric variables were recorded, as well as the animal’s sex. Four algorithms—linear discriminant analysis, quadratic discriminant analysis, logistic regression, and classification trees—were used to classify the breeds. The results indicated that head width, height at the withers, and right ear length measurements could be used to differentiate the “Casco de Mula” and San Pedreño Creole pigs. The decision tree was the most accurate algorithm (accuracy = 92%, sensitivity = 96%, specificity = 83%, and Matthews correlation coefficient = 0.82), and its performance can be improved by increasing the number of animals. Non-parametric supervised learning methods like decision trees can be used to morphometrically differentiate Creole pigs raised in the same or different environments in order to characterize animal genetic resources.

## 1. Introduction

Creole pigs (*Sus scrofa domestica*) are descended from Iberian pigs that were brought by Cristobal Colon on his second voyage (1493), arriving at the island of Hispaniola, to subsequently be introduced to the American continent (1495) [1]. These animals, with possible origin in the Iberian and Celtic breeds [2], served as initiators of the animal industry in different colonies formed in the New World [3,4]. Creole pigs have usually been managed traditionally and mostly with no special facilities, which has made them recognized for their lower productivity compared to improved pigs raised in more technical production systems [1,5]. This genetic resource has been undervalued, and efforts for its conservation and use have been limited [6].

In Latin America, Creole pigs are diverse and genetically similar, with an important cultural heritage. The Creole breeds are highly adaptable to different ecological conditions with a natural capacity to take advantage of the available resources. These characteristics make them a valuable reservoir of genetic variability useful for sustainable exploitation or to be used in crosses with commercial breeds [7]. Adaptive characteristics are especially important, since adverse environmental conditions could lead to morphometric variations within the populations, with distinctive adaptation traits between races, useful for consideration in recovery and preservation programmes [8,9,10].

In Colombia, the San Pedreño (SP) and “Casco de Mula” (CM) Creole pigs are commonly found, especially in small-scale production systems, representing an important source of energy, protein, and income for rural family units [1,11]. Nowadays, these Creole breeds are underutilized and at risk of disappearing due to the preferential use of foreign improved breeds in the national swine industry [12,13]. In the last decade, this situation has motivated increased awareness about the importance of preserving these genetic resources, in order to maintain the tradition and culture of rural families [5]. Both breeds represent a heritage of biodiversity, since they have lived and naturally adapted to different environments and nutritional limitations [11].

**Figure 1:**
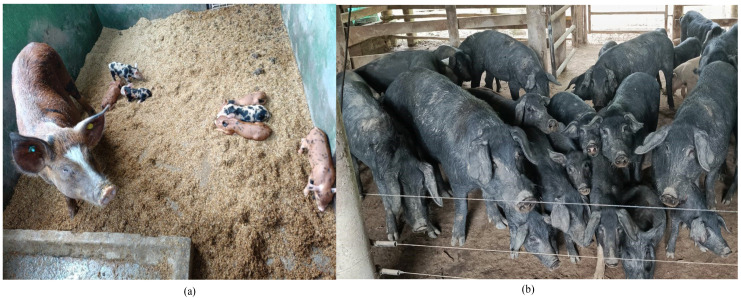
Colombian Creole pigs. (**a**) Casco de Mula; photography: CI Agrosavia. (**b**) San Pedreño; photography: Casa Blanca Farm.

The “Casco de Mula” pig is found in the Eastern Plains and the foothills of the Llanos of Colombia (Figure 1a). This breed is medium-sized, with black skin and red or black hair. It has a concave profile, with large ears that fall slightly forward. The legs are strong and short and present syndactyly or union of the toes [4,14]. The San Pedreño pig (Figure 1b) can be found in several regions of Colombia and is characterized by a concave profile, small head, straight and medium-sized ears, and abundant black hair [4].

Racial characterization based on morphometry and morphology is one of the first phases for maintenance, conservation, and knowledge of local zoogenetic resources [15,16]. Animals’ morphological structure and functional qualities define the population characteristics, mark the productive, functional, and ethnological trends of the breeds [5,16,17], and allow phenotypic comparisons between domestic species of different breeds in different environments [17,18].

In recent years, morphometric techniques associated with multivariate statistical analysis have been shown to be useful for the study of animal’s phenotypic characteristics [18,19]. Classical statistical techniques, many of them now used in machine learning (ML) to fit data either for regression or classification analysis, have proven to be efficient for morphometric analysis in different scientific disciplines [19,20,21,22]. White-box supervised multivariate statistical algorithms, such as linear discriminant analysis, quadratic discriminant analysis, logistic regression, and classification trees, are commonly used for binary classification. In the first method, the covariance matrices within groups are assumed to be homogeneous. Even when this assumption is not true, it is still possible to develop linear functions with good classification capabilities or to try to fit quadratic functions to improve the classification task. In the logistic regression and classification trees, greater flexibility can be found, since it is possible to work with non-normal and even categorical characteristics. In animal science, multivariate algorithms are increasingly used to develop models in different areas such as animal nutrition, health, welfare, and reproduction [23]. The algorithms have been used to predict body weight in pigs and foals [23,24], the results of horse racing [25], mastitis infections [26], clinical ketosis [27], heat stress in cows [28], and parturition events in grazing sheep [29]. It can be generally assumed that logistic regression and discriminant analysis are more “statistical” methods, and trees can be placed closer to machine learning. Considering the importance of multivariate statistical methods to adjust high-performance predictive models, the aim of this study was to identify the most representative body morphometric measurements to differentiate two Colombian Creole pig breeds (“Casco de Mula” and San Pedreño) using statistical and machine learning methods.

## 2. Materials and Methods

### 2.1. Study Site

This study was carried out in the Meta department, Colombian Orinoquia (latitude: 01°36′29″ and 04°54′24″ N; longitude 71°04′42″ and 74°54′09″ W). The department is located in the tropical zone, with a flat and undulating topography, at an altitude between 200 and 450 m, and annual precipitation ranging between 2700 and 3500 mm. The average ambient temperature and relative humidity were 26 °C and 71%, respectively [30,31].

### 2.2. Animals Sampled

Body morphometric measurements of 84 Creole pigs (54 “Casco de Mula”-CM and 30 San Pedreño-SP) were taken, including both sexes and with ages between 2 and 6 months. The animals belonged to seven different traditional farms selected for convenience. Data collection lasted 12 months (September 2023 to September 2024).

### 2.3. Measured Variables

In total, 14 body measurements were recorded, including body weight (BW), thoracic perimeter (TP), body length (BL), height at the withers (HaW), sternum height (SH), knee perimeter (KP), head length (HL), head width (HW), left ear length (LEL), left ear width (LEW), right ear length (REL), right ear width (REW), posterior cannon perimeter (PCP), and hock height (HH). Body measurements were taken with a swine measuring tape and body weight with a digital hanging scale (Penesanio, CO). All measurements were taken by a single person, after training on the anatomical points for measurements.

### 2.4. Statistical Analysis

The morphometric variables were subjected to descriptive statistics, the Shapiro– Wilk normality test, and Spearman correlations (S_c_). Statistical significance was declared at *p* ≤ 0.05. In highly correlated variables (>0.90), only one was chosen for further analysis to avoid multicollinearity effects. Four white-box multivariate statistical algorithms (linear discriminant analysis—LDA; quadratic discriminant analysis—QDA; logistic regression—LR; and classification trees—CTs) were analysed for the classification of the “Race” target variable (“Casco de Mula” or San Pedreño) based on the corporal morphometric characteristics, including the sex effects.

In the LDA and QDA analyses, variables were transformed (log_10_ or square root) to ensure an approximately normal distribution, when necessary. Later, they were centred and scaled. In these algorithms, thirteen morphometric characteristics (TP, BL, HaW, SH, KP, HL, HW, LEL, LEW, REL, REW, PCP, and HH) were used to predict the “Race” target variable. In the LR and CT algorithms, the same model mentioned above (with the variables on their original scale) plus the sex variable was used. In the LR, only significant characteristics (*p* < 0.05) were retained in the final model, while in the CT, the classification and regression tree algorithm (CART) was used, and pruning to reduce model complexity was performed, using the “complexity parameter (CP)” criterion, which imposes a penalty on trees with many branches. Ten CP values were evaluated, and the most accurate was estimated using 10-fold cross-validation.

The models were subjected to leave-one-out cross-validation, and their performance was analysed by constructing confusion matrices and estimating the accuracy (proportion of correct predictions in both races), sensitivity (proportion of CM animals correctly classified among the observed CM sample), specificity (proportion of SP animals correctly classified among the observed SP sample), precision or positive predictive value (proportion of CM animals correctly classified among the predicted CM animals), negative predictive value (NPV; proportion of SP animals correctly classified among the predicted SP animals), model error rate, and the Matthews correlation coefficient to account for the imbalance in the data.

Receiver operating characteristic curves (ROC curves) were created, and the areas under the curve (AUCs) were estimated. With the best classification model, the cut-off point with the highest sensitivity and specificity were obtained. Analyses were performed in Rstudio 4.3.0, using the Mass, Caret, rpart, pRoc, and boot libraries [32].

## 3. Results

Descriptive statistics of the morphometric variables are shown in Table 1. Most of the characteristics presented an acceptable variation (CV = 11.5 to 25.6%), except for body weight (CV = 73.8% on average for both races) and posterior cannon perimeter in the “Casco de Mula” animals (CV = 43.3%). The other morphometric measurements were similar between breeds. The Shapiro–Wilk test indicated that 11 of the 14 morphometric variables (BW, TP, BL, KP, HL, HW, LEW, REL, REW, PCP, and HH) were not normally distributed (*p* < 0.05). The Spearman correlation analysis showed that BW and TP characteristics were positively associated (r_s_ = 0.95; *p* < 0.0001); so, to avoid multicollinearity, only TP was selected for further analyses, since BW was highly variable. In this way, 13 morphometric variables and the sex effect were used for analysis.

Confusion matrices and model performance measurements during the leave-one-out cross-validation are presented in Table 2 and Table 3, respectively. According to the classical confusion matrix parameters, the studied models classified both breeds appropriately, with proportions of correct predictions (accuracy) between 0.79 and 0.92, and errors ranging between 0.08 and 0.21. The model with the best performance was the CT, with a proportion of correct predictions of 92%, and it was able to adequately classify the “Casco de Mula” and San Pedreño pigs with a sensitivity of 96% and a specificity of 83%, respectively. To assess the model’s classification considering the imbalance of the data (CM = 54 and SP 30), the MCC was estimated, which ranged from 0.53 to 0.82 between models, with the CT algorithm performing best.

**Figure 2:**
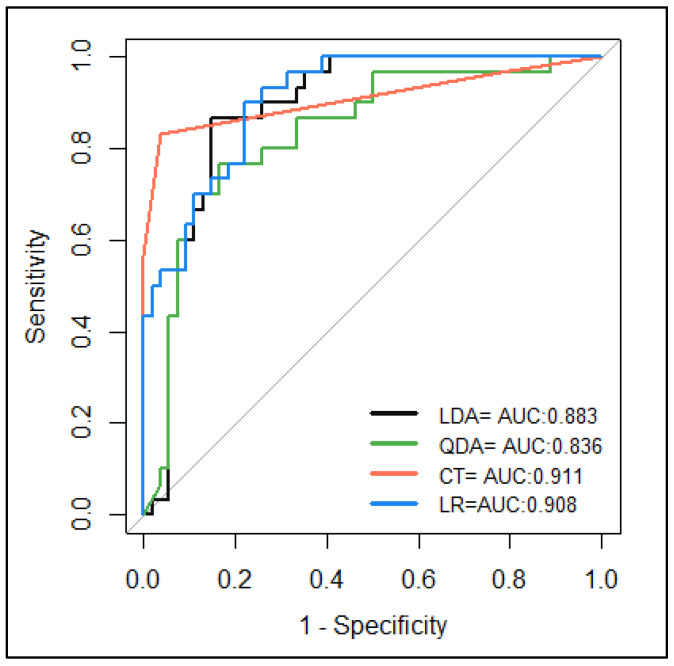
The ROC curve and area under the curve (AUC) of the studied models. LDA: linear discriminant analysis; QDA: quadratic lineal discriminant analysis; LR: logistic regression; CT: classification tree.

The ROC curves of the models and their respective AUCs are shown in Figure 2. This graph relates sensitivity vs. 1-specificity and allowed us to analyse the trade-off between correct and incorrect predictions. The areas under the curve (AUCs) were used to summarize the model’s performance during the classification, considering all the possible probability thresholds or cut-offs, and served to evaluate the model’s discriminatory capacity between “Casco de Mula” and San Pedreño animals. The higher the AUC values (>0.80), the better the classification capacity of the model [33]. The results showed that all models presented high classification capacities, with AUC values ranging from 0.836 to 0.911. Among the studied algorithms, the CT model was the best-performing, reaching maximum values of sensitivity and 1-specificity of 0.83 and 0.037, respectively, with a cut-off point of 0.55 (Figure 2).

**Figure 3:**
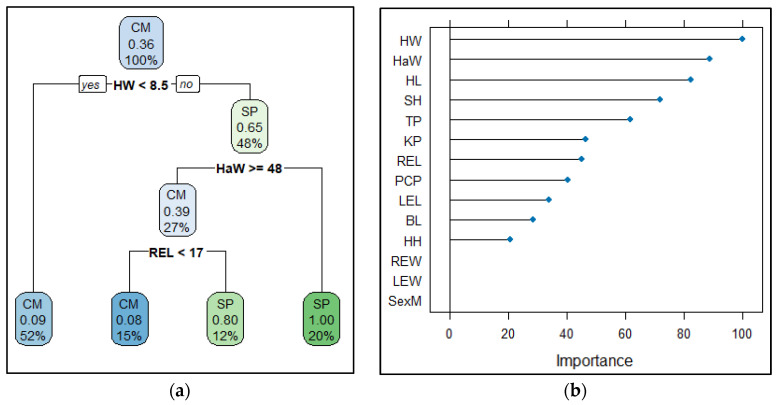
(**a**) Final classification tree. Information within nodes from top to bottom corresponds to predictions, predicted probability of SP breed, and percentage of observation in the node, respectively. (**b**) Variable importance graph. CM: “Casco de Mula”; SP: San Pedreño; TP: thoracic perimeter; BL: body length; HaW: height at withers; SH: sternum height; KP: knee perimeter; HL: head length; HW: head width; LEL: left ear length; LEW: left ear width; REL: right ear length; REW: right ear width; PCP: posterior cannon perimeter; HH: hock height.

The resulting classification tree with the ability to discriminate between “Casco de Mula” and San Pedreño Creole pigs, as well as the most important variables to its construction, is shown in Figure 3.

The most important morphometric measurements that contributed to differentiate the Creole pigs were head width (HW; 100%) and height at the withers (HaW; 89.02%). The right ear length (REL) variable had a 45.12% importance and, together with HW and HaW, improved the Creole pig’s classification. Figure 3 shows the description of the classification tree for the “Casco de Mula” and San Pedreño Creole pig breeds.

## 4. Discussion

Morphometric analysis provides information on the species morphological structure and the factors influencing their functional abilities and ethnological characteristics [34,35]. This information is required to define the species in the face of geographic dispersion and sexual differentiation [36]. In commercial scenarios, morphometric measurements as well as animal productive records constitute the base information to identify populations, races, and animals’ productive potential, in order to efficiently use the porcine genetic resources [37,38].

In the present study, the morphometric measurements that contributed to classifying the “Casco de Mula” and San Pedreño Creole pigs were the head width (HW), height at the withers (HaW), and right ear length (REL). The San Pedreño animals showed greater HW and REL, while the HaW was slightly higher in the “Casco de Mula” animals. These differences can be related to age differences among the sampled animals in both breeds, similar to what was reported in Creole pigs from Ecuador [39], Ch’orti pigs from Guatemala [40], and the Pampa Rocha pig from Uruguay [41]. The head width (HW) and right ear length (REL) are measurements related to ethnological characters and are important in the characterization and comparison between breeds, as well as to establish differences between sexes [42].

In the Casco de Mula and San Pedreño Creole breeds, the head width values were less than those reported in Creole pigs from the province of Loja, Ecuador [39], Ch’ortí pigs from Guatemala [40], Creole pigs from Nicaragua [43], Pampa Rocha pigs from Uruguay [41], Venezuelan Creole pigs [7], Pillareño pigs from Ecuador [44], pigs from Chocó, Colombia [45], and Cuban Creole pigs [46]. However, this cephalic measurement was greater than the value reported for the Araucanian Creole pig [47].

Ear length is a trait that correlates with the animal profile. The “Casco de Mula” and San Pedreño pigs have a concave profile, meaning that their ears are large and at the same time fall to the sides or forward. This contrasts with subconcave profiles, where the ears are medium-sized and point forward in an almost horizontal direction, and ultra-concave profiles, where the ears are small and point upwards [42]. The ear length of the San Pedreño and “Casco de Mula” pigs was shorter than the values reported for other Ibero-American Creole pigs [39,41,44,45], except for the Araucanian Creole pig [47].

The height at the withers is a measure that determines the animal size and is useful to define lines within breeds [48]. This character is almost not influenced by environmental conditions, so it is suitable to delimit differences or similarities between pigs of different origins [49]. Since the height at the withers is not associated with ethnological characters, the observed differences between Creole pigs could be attributed mainly to breeding and artificial selection rather than the ecological conditions of the region they inhabit. The height at the withers of “Casco de Mula” and San Pedreño animals was lower compared to other Ibero-American Creole pigs from Mexico [37,50], Ecuador [39,44], Nicaragua [43], Uruguay [41], Venezuela [7], Colombia [45], and Cuba [46]. This body measurement was higher compared to the Ch’ortí pig from Guatemala [40] and similar to the values reported for the Araucanian Creole pig [47].

According to the accuracies values obtained (0.79 to 0.92), the multivariate statistical models used in the present study classified both Creole pigs appropriately. These accuracies are similar to the 0.77 to 0.96 values reported in other studies, where algorithms such as support vector machine, decision tree, linear discriminant analysis, and artificial neural networks have been successfully used for population classification of carp [51], fruit fly species [52], and mosquito species [53].

The decision tree algorithm was the best classification model with the observed and predicted data. The model showed the lowest error rate, and according to the sensitivity and specificity, 96% and 83% of the CM and SP animals, respectively, were correctly classified in the observed data, while the precision and NPV suggested greater model efficiency with predicted data, since 91% and 93% of the CM and SP animals, respectively, were correctly classified. Theses metrics suggest an optimal performance of the DT model; however, imbalance between categories must be considered. The MCC is useful since it accounts for all the entries of the confusion matrix and provides a robust metric to evaluate the classifiers performances. MCC values close to 1 indicate a perfect classification, 0 is a random classification, and −1 indicates disagreement between the observed and predicted categories [54]. In the study, the highest MCC was also observed in the DT algorithm (0.82), nonetheless with a lower value than the other metrics, indicating that data imbalance influenced the model performance. An MCC of 0.82 is still close to 1, which allows a strong association between the observed and predicted categories to be inferred.

The decision tree algorithm has also been used successfully in a carp classification study [51]. This model is often used in biological data processing for its simplicity, interpretability, and the ability to provide solutions in classification, prediction, and pattern identification processes [51]. This result suggests that in morphometric studies that aim to characterize animal genetic resources, besides the use of traditional statistical methods such as the linear models, machine learning algorithms constitute another alternative to address this issue, providing in some situations more consistent, accurate, interpretable, and automated results than the standard geometric morphometric analysis [55]. The results of the present study constitute one of the first efforts to characterize and differentiate the “Casco de Mula” and San Pedreño Creole pigs, considering that it is difficult to carry out this type of research since, as they are animals at risk, their population is small and difficult to find. It is hoped that further, similar studies will be carried out, hopefully with a larger sample size.

## 5. Conclusions

This study indicates that “Casco de Mula” and San Pedreño Creole pigs could be differentiated using morphometric measures such as head width, height at the withers, and right ear length measurements. Studies with a larger sample size are required to confirm these results. The decision tree was the most accurate algorithm, and its performance was consistent even with unbalanced data. Non-parametric supervised learning methods like decision trees can be used to morphometrically differentiate Creole pigs raised in the same or different environments, in order to characterize the animals’ genetic resources.

## Figures and Tables

**Table 1:** Descriptive statistics of body morphometry (cm) from “Casco de Mula” and San Pedreño Creole pigs. Body weight is given in kg.

Creole Pig	Variable	Mean	SD	CV (%)	Minimum	Maximum
“Casco de Mula”	BW (kg)	30.4	21.9	72.0	7.1	150.0
	TP (cm)	69.4	14.6	21.0	44.0	131.0
	BL (cm)	58.2	11.9	20.4	38.0	120.0
	HaW (cm)	48.6	8.7	17.9	31.0	78.0
	SH (cm)	24.8	4.5	18.3	12.0	34.0
	KP (cm)	15.0	2.8	18.9	9.0	25.0
	HL (cm)	21.6	3.8	17.5	15.0	40.0
	HW (cm)	8.3	1.9	22.4	5.5	17.0
	LEL (cm)	15.1	2.6	17.4	9.9	22.0
	LEW (cm)	10.6	2.0	19.0	7.0	19.0
	REL (cm)	15.1	2.5	16.6	10.0	23.0
	REW (cm)	10.5	1.9	18.1	7.0	16.0
	PCP (cm)	16.0	6.9	43.3	10.0	62.0
	HH (cm)	19.4	2.9	15.0	15.0	29.0
San Pedreño	BW (kg)	40.8	30.8	75.6	14.7	125.0
	TP (cm)	75.1	17.3	23.0	57.0	120.0
	BL (cm)	63.1	13.5	21.4	41.0	91.0
	HaW (cm)	47.2	12.0	25.4	14.0	68.0
	SH (cm)	22.0	5.6	25.6	12.5	33.0
	KP (cm)	18.3	4.3	23.4	13.0	31.0
	HL (cm)	23.5	4.4	18.7	17.0	33.0
	HW (cm)	9.9	1.3	13.0	7.0	13.0
	LEL (cm)	15.9	3.2	19.8	10.0	22.0
	LEW (cm)	11.4	1.7	15.0	9.0	17.0
	REL (cm)	16.0	3.3	20.9	11.0	23.0
	REW (cm)	11.6	1.7	14.7	9.0	17.0
	PCP (cm)	17.7	3.8	21.6	13.0	28.0
	HH (cm)	19.7	2.3	11.5	16.0	23.0

SD: standard deviation; CV: coefficient of variation; BW: body weight; TP: thoracic perimeter; BL: body length; HaW: height at withers; SH: sternum height; KP: knee perimeter; HL: head length; HW: head width; LEL: left ear length; LEW: left ear width; REL: right ear length; REW: right ear width; PCP: posterior cannon perimeter; HH: hock height.

**Table 2:** Confusion matrices obtained with the studied models during the leave-one-out cross-validation.

LDA	Observed		LR	Observed	
Predicted	CM	SP	Predicted	CM	SP
CM	46	7	CM	48	10
SP	8	23	SP	6	20
**QDA**	**Observed**		**CT**	**Observed**	
Predicted	CM	SP	Predicted	CM	SP
CM	45	9	CM	52	5
SP	9	21	SP	2	25

CM: “Casco de Mula”; SP: San Pedreño; LDA: linear discriminant analysis; LR: logistic regression; QDA: quadratic discriminant analysis; CT: classification tree.

**Table 3:** Performance measurements of the studied models during the leave-one-out cross-validation.

Model	LDA	QDA	LR	CT
Accuracy	0.82	0.79	0.81	0.92
Sensitivity	0.85	0.83	0.89	0.96
Specificity	0.77	0.70	0.67	0.83
Precision	0.87	0.83	0.83	0.91
NPV	0.74	0.7	0.77	0.93
MCC	0.61	0.53	0.58	0.82
Error rate	0.18	0.21	0.19	0.08

LDA: linear discriminant analysis; QDA: quadratic discriminant analysis; LR: logistic regression; CT: classification tree; MCC: Matthews correlation coefficient. NPV: Negative predictive value.

## Data Availability

The data presented in this study are available on request from the second author. The data have not been published because we are going to carry out further analysis.
